# Multidrug-Resistant Shigellosis among Children Aged below Five Years with Diarrhea at Banadir Hospital in Mogadishu, Somalia

**DOI:** 10.1155/2021/6630272

**Published:** 2021-06-08

**Authors:** Bilan Sheikh Ali Nor, Nelson Chengo Menza, Abednego Moki Musyoki

**Affiliations:** ^1^Department of Medical Laboratory Sciences, Kenyatta University, Nairobi, Kenya; ^2^Department of Pediatrics, Banadir Hospital, Mogadishu, Somalia; ^3^National Public Health Reference Laboratory (NPHRL) in Mogadishu, Mogadishu, Somalia

## Abstract

Globally, shigellosis remains the second leading cause of diarrhea-associated deaths among children under five years of age, and the infections are disproportionately higher in resource-limited settings due to overcrowding, poor sanitation, and inadequate safe drinking water. The emergence and global spread of multidrug-resistant (MDR) *Shigella* are exacerbating the shigellosis burden. We adopted a cross-sectional study design to determine the distribution and antimicrobial susceptibility (AST) patterns of *Shigella* serogroups among children aged below five years presenting with diarrhea at Banadir Hospital in Mogadishu, Somalia, from August to October 2019. Stool and rectal swab samples were collected from 180 children consecutively enrolled using a convenient sampling technique and processed following standard bacteriological methods. AST was determined using the Kirby–Bauer disc diffusion method and interpreted as per the Clinical Laboratory Standard Institute (2018) guidelines. Shigellosis prevalence was 20.6% (37/180), and *S. flexneri* (26/37 (70.3%)) was the predominant serogroup. All the serogroups were 100% resistant to ampicillin (AMP), trimethoprim-sulfamethoxazole (SXT), and tetracycline (TE). Ceftriaxone (CRO) resistance was the highest among *S. sonnei* (66.7%) isolates. 19.2% of *S. flexneri* and *S. sonnei* (50%) serogroups were resistant to ciprofloxacin (CIP), but all *S. dysenteriae* type 1 isolates remained (100%) susceptible. Forty percent of CIP-susceptible *S. dysenteriae* type 1 were resistant to CRO. Seven MDR *Shigella* phenotypes were identified, dominated by those involving resistance to AMP, SXT, and TE (100%). Our findings showed a high prevalence of shigellosis with *S. flexneri* as the most predominant serogroup among children under five years of age in Banadir Hospital, Somalia. AMP and SXT are no longer appropriate treatments for shigellosis in children under five years in Banadir Hospital. MDR *Shigella* strains, including those resistant to CIP and CRO, have emerged in Somalia, posing a public health challenge. Therefore, there is an urgent need for AMR surveillance and continuous monitoring to mitigate the further spread of the MDR *Shigella* strains in Banadir Hospital and beyond.

## 1. Introduction

Globally, approximately 165 million episodes of bacillary dysentery (shigellosis) and about 1.1 million associated deaths occur annually [1]. Developing countries, whereby 69% of the episodes and 61% of deaths occur among children under five years, bear the highest burden of these infections (99%) [2]. This disproportionate shigellosis burden is attributable to overcrowding, poor sanitation, and limited access to safe drinking water [2, 3]. The causative agent of bacillary dysentery is *Shigella* bacteria. They are facultative anaerobes, nonmotile, and none spore-forming Gram-negative microorganisms. Serologically the pathogen is grouped into four distinct serogroups, namely *Shigella dysenteriae* (Shiga bacillus), *S. flexneri, S. boydii*, and *S. sonnei*. The distribution of these serogroups varies geographically, whereby *S. flexneri* and *S. sonnei* are predominant in developing and developed countries, respectively [1].

Even though most cases of dysentery are usually self-limiting, appropriate antimicrobial therapy prescription is required to improve the clinical outcome of the disease, decrease the period of illness, and reduce bacterial shedding time [4]. Ciprofloxacin is the World Health Organization (WHO) first-line treatment option for dysentery. Pivmecillinam, ceftriaxone, or azithromycin (in adults only) are the second-line treatment options [2]. However, *Shigella* strains resistant to these antibiotics are increasingly emerging and spreading globally, thus posing a serious public health threat [5].

In Somalia, three-quarters of the population lacks sanitary facilities, 45% have no access to clean and safe drinking water, and about 3.2 million lack access to healthcare facilities [6, 7]. Currently, the country is facing a public health crisis due to civil war and political unrest that have been ongoing for decades affecting the establishment of functional healthcare service delivery systems. Also, the poorly managed and overcrowded internally displaced persons (IDP) camps with no access to adequate safe drinking water and sanitary facilities serve as hotspots for diarrhea disease outbreaks. As per the findings of a study carried out by Global Burden of Disease (1990 to 2015), Somalia had the highest diarrhea mortality among children under five years in Eastern Mediterranean Region, and shigellosis was the second topmost killer [6]. Unpublished data in Somalia show that the traditional first-line empiric therapy antibiotics, including co-trimoxazole and ampicillin, are used in shigellosis treatment, against the WHO guidelines. This poses a serious public health concern due to multidrug-resistant infections. Yet, current epidemiological data on bacillary dysentery in Somalia are limited. Scientifically based information is critical for defining an appropriate empiric therapy and preventing the emergence and spread of multidrug-resistant shigellosis in Somalia. Therefore, this study aimed to determine the distribution and antimicrobial susceptibility patterns of *Shigella* serogroups among children of less than five years presenting with diarrhea at Banadir Hospital in Mogadishu, Somalia.

## 2. Methods

### 2.1. Design

A cross-sectional study to determine the distribution and antibiotic susceptibility of *Shigella* serogroups among children under five years was carried out at Banadir Hospital from August to October 2019.

### 2.2. Setting and Sample Collection

Banadir Hospital is located in Wadajir District, South West of the Banadir region of Somalia. It is the largest mother and child hospital (MCH) in Mogadishu, Somalia. The study excluded children with a history of antibiotic use within two weeks before the sample collection date. A structured questionnaire was completed for each participant through a face-to-face interview to assess the factors associated with shigellosis. A total of 180 samples, including 34 stool and 146 rectal swab samples, were collected from children with clinically diagnosed diarrhea. We collected stool samples in sterile screw-cap containers with Cary Blair Transport Medium (Medical Chemical Corporation, Torrance, CA, USA) [8]. In cases where stool samples were not available, rectal swabs were collected and immediately placed in the Cary Blair Transport Medium. The samples were then transported in an icebox to the National Public Health Reference Laboratory (NPHRL) in Mogadishu, Somalia, for analysis within 2-3 hours.

### 2.3. Bacterial Isolation, Biochemical Characterization, and Serogrouping

We isolated and identified *Shigella* as per the standard bacteriological methods [9, 10]. Samples were aseptically inoculated into selenite F broth (Oxoid, UK) and incubated aerobically at 37°C for 18–24 hours; subsequently, a loopful of the culture was streaked on MacConkey agar (Merck, Germany) and xylose lysine deoxycholate (XLD) agar (Oxoid, UK) and incubated aerobically at 37°C for 18–24 hours. Biochemical tests including carbohydrates fermentation (triple sugar iron agar), urea test, indole test, and motility test were then used to identify the resultant colonies. *Shigella* serogroups were determined using a commercially available serological kit (Denka Seiken, Tokyo, Japan).

### 2.4. Antimicrobial Susceptibility Testing

The choice of the antibiotics tested in this study was guided by clinical data on drugs used in the management of diarrheal diseases in Banadir Hospital, Somalia. To this end, we tested the antimicrobial susceptibility of *Shigella* serogroups to six antibiotics: including ampicillin (10 *μ*g), tetracycline (30 *μ*g), trimethoprim-sulfamethoxazole (1.25 *μ*g), ciprofloxacin (30 *μ*g), ceftriaxone (30 *μ*g), and azithromycin (15 *μ*g) (Oxoid, UK) using the Kirby–Bauer disc diffusion method. A bacterium suspension equivalent to 0.5 McFarland standard was prepared and inoculated on Mueller–Hinton agar (Merck, Germany). The plates were allowed to dry for 3–5 minutes, antimicrobial discs were placed on the medium, and subsequently, the culture plates were incubated at 37°C for 24 hours. The minimum inhibition zones were determined and interpreted following the Clinical Laboratory Standards Institute (CLSI, 2018) [11] guidelines, and *Escherichia coli* strain (ATCC 25922) was used as a quality control organism.

### 2.5. Data Analysis

The obtained data were analyzed using IBM SPSS version 23 software. Descriptive data were generated and presented in tables, and the Chi-square (*X*^2^) statistic was used to test the relationship between the occurrence of shigellosis and the patient's sociodemographic characteristics and environmental conditions. Variables showing a significant relationship using *X*^2^ were further analyzed using Pearson correlation statistics. A *p* value less than 0.05 was considered statistically significant.

## 3. Results

### 3.1. Sociodemographic Characteristics of Children and Caregivers

We sampled 180 children under the age of five presenting with diarrhea at Banadir Hospital, Somalia. Of these, 69/180 (38.3%) and 111/180 (61.7%) were male and female, respectively. The mean age of the children was 20.1 months (standard deviation of 12.87 months). Based on the age, we categorized the children into five groups, with the majority under the age group >12–24 months (43.3%) and living in households with two children (48.9%). Most of the caregivers aged between 25 and 30 years (41.1%), were married (74.4%), and had no formal education (78.4%) (Table 1).

### 3.2. Prevalence of Shigellosis and *Shigella* Serogroups' Distribution among Children under Five

The overall prevalence of shigellosis among children presenting with diarrhea at Banadir Hospital was 20.6% (37/180). We isolated three *Shigella* serogroups: serogroup A (*Shigella dysenteriae* type 1), B (*S. flexneri*), and C (*S. sonnei*). Serogroup D (*S. boydii*) was absent*. Shigella flexneri* (26/37, 70.3%) was the most predominant serogroup, followed by *S. sonnei* (6/37, 16.2%) and *S. dysenteriae* (5/37, 13.5%) (*t* = 15.187; *p*=0.0001).

### 3.3. Antimicrobial Susceptibility Patterns of *Shigella* Serogroups

All the isolated *Shigella* serogroups were 100% resistant to ampicillin, trimethoprim-sulfamethoxazole, and tetracycline (Table 2). Additionally, 19.2% *S. flexneri* and *S. sonnei* (50%) were resistant to ciprofloxacin, but *S. dysenteriae* type 1 remained susceptible. However, 40% of the ciprofloxacin-susceptible *S. dysenteriae* type 1 were ceftriaxone resistant. The highest resistance to ceftriaxone occurred among *S. sonnei* (66.7%) serogroup, followed by *S. dysenteriae* type 1 (40%) and *S. flexneri* (38.5%). None of the serogroups was resistant to azithromycin (Table 2).

Based on resistance to two or more classes of antibiotics, we categorized the *Shigella* serogroups into seven multidrug-resistant (MDR) phenotypes. The most dominant phenotype was *AMP/SXT/TE* (37/37, 100%), followed by *AMP/SXT/CRO or SXT/TE/CRO* (6/37, 42.2%)*, AMP/SXT/TE/CRO* (13/37, 35.1%)*, AMP/SXT/CIP or SXT/TE/CIP* (8/37, 21.6%)*, AMP/SXT/TE/CIP* (5/37, 13.6%), and *AMP/SXT/TE/CIP/CRO* (3/37, 8.1%). MDR phenotypes of *S. dysenteriae* type 1 (*AMP/SXT* or *AMP/TE* or *SXT/TE*, and *AMP/SXT/TE*) were susceptible to ciprofloxacin, but 50% (3/6) of *S. sonnei* isolates and *S. flexneri (*19.5%, 5/26) remained resistant. Among the serogroups, most of the MDR phenotypes were found in *S. flexneri* (65.9%), followed by *S. sonnei* (20.7%) and *S. dysenteriae* type 1 (13.4%) isolates.

### 3.4. Factors Associated with Shigellosis among Children under Five Years of Age

Even though *Shigella* infections appeared to be higher among children in the age group >12–24 months (43.3%) (*χ*2 = 10.601, *p* value = 0.031) whose caregivers had no formal education (*χ*2 = 13.515; *p*=0.004), correlation statistical analysis showed no such relationship (Table 3).

## 4. Discussion

We investigated the distribution and antibiotic susceptibility of *Shigella* among children under the age of five years in Somalia, a country with the highest burden of diarrhea mortality among children less than five years in the Eastern Mediterranean Region [6]. The overall prevalence of shigellosis in Banadir Hospital was 20.6%, higher than 4.0%–17.8% documented in Kenya [12], Ethiopia [13, 14], Tanzania, South Africa, Pakistan, Nepal, Peru, Bangladesh, Brazil, and India [15]. This discrepancy suggests poor sanitary conditions, limited access to safe drinking water, and poor hygiene in Somalia. Moreover, the decades-long civil and political unrest in Somalia has negatively impacted healthcare service delivery and rehabilitation of drinking water and sewage systems. According to the United Nations Children's Fund (UNICEF), three-quarters of Somalia population lacks sanitary facilities, and 45% have no access to safe drinking water in Somalia [7–16].

Three *Shigella* serogroups, serogroup A (*Shigella dysenteriae* type 1), B (*S. flexneri*), and C (*S. sonnei*), caused bacillary dysentery among children under five years at Banadir Hospital, Somalia. Serogroup *D* (*Shigella* boydii) was absent, an unsurprising finding since this bacterium is more prevalent in Southeast Asia (Nepal and Bangladesh) [17] but rarely reported elsewhere in the Central African Republic [18], Ethiopia, and Egypt [19]. In our study, the most prevalent serogroup was *Shigella flexneri* (70%), a finding consistent with other reports from Nigeria [20], Central Africa Republic [18], Nepal [21], Iran, India, and Eritrea [22, 23]-(4). Epidemiologically, *Shigella* serogroups show geographical stratification based on the economic and development status of a country, as well as the virulence of a given serogroup. Contrary to the developed world, *Shigella flexneri* is most predominant in developing countries where people are likely to have acquired immunity to *S. sonnei* following frequent exposure to *Plesiomonas shigelloides* in faecally contaminated water [24].

The emergence and global spread of multidrug-resistant (MDR) strains continue to pose serious public health problems and complicate the selection of appropriate empirical therapy [25]. In this study, all the *Shigella* serogroups were 100% resistant to ampicillin (AMP), trimethoprim-sulfamethoxazole (co-trimoxazole, SXT), and tetracycline (TE). To the best of our knowledge, this was the highest *Shigella* resistance level to these three antibiotics, not only in Somalia but also in the neighboring countries. Resistance ranging from 80% to 100% has been reported in Kenya [26], Central Africa Republic [18], and Iran [27, 28]. AMP, SXT, and TE are low-cost antibiotics available over-the-counter and often used as empiric therapy for diarrheal cases in many developing countries and, therefore, the observed high-level resistance may have arisen due to overuse or misuse of these drugs. Our finding suggests that AMP and SXT are no longer appropriate empiric therapy for the treatment of bacillary dysentery among children under five years in Banadir Hospital, Somalia.

The current World Health Organization (WHO) guidelines for the treatment of shigellosis recommends ciprofloxacin as a first-line option or either Pivmecillinam, ceftriaxone, or azithromycin (in adults only) as second-line antibiotics [2]. In this study, ciprofloxacin (CIP) was only effective against *S. dysenteriae* type 1 serogroup, but some strains of *S. flexneri* (19.2%) and *S. sonnei* (50%) remained resistant. These findings are inconsistent with Liu and colleagues report, documenting CIP resistance of 49.7% and 19.1% for *S. flexneri* and *S. sonnei*, respectively [29]. In the current study findings, 40% of the CIP-susceptible *S. dysenteriae* type 1 were ceftriaxone (CRO) resistant, and the highest CRO resistance occurred among *S. sonnei* (66.7%) serogroup, followed by *S. dysenteriae* type 1 (40%) and *S. flexneri* (38.5%). The CRO resistance was higher than 33.8% for *S. sonnei* and lower than 45.7% for *S. flexneri* reported in China [29]. Our study findings suggest a diminishing clinical value of CIP and CRO in Somalia, posing serious public health concern due to drug-resistant *Shigella* infections.

In the current study, none of the serogroups was resistant to azithromycin (AZM), which corroborates with other findings elsewhere in Iran and the USA [27–30] but inconsistent with reports that documented decreasing susceptibility to AZM in India (48%) [31], Palestine (42%) [32], and Australia (13.1%) [33]. This discrepancy may be due to differences in the composition of study populations, where the current study focused only on children under five years. WHO does not recommend AZM for bacillary dysentery in children under five years, which may account for the absence of resistance observed in the current study.

Based on resistance to two or more classes of antibiotics, we identified seven multidrug-resistant (MDR) phenotypes, dominated by AMP/SXT/TE (37/37, 100%), followed by AMP/SXT/CRO or SXT/TE/CRO (6/37, 42.2%), AMP/SXT/TE/CRO (13/37, 35.1%), AMP/SXT/CIP or SXT/TE/CIP (8/37, 21.6%), AMP/SXT/TE/CIP (5/37, 13.6%), and AMP/SXT/TE/CIP/CRO (3/37, 8.1%). MDR phenotypes of *S. dysenteriae* type 1 (AMP/SXT or AMP/TE or SXT/TE, and AMP/SXT/TE) were susceptible to ciprofloxacin, but 50% (3/6) of *S. sonnei* isolates and *S. flexneri* (19.5%, 5/26) remained resistant. These profiles are similar to those reported in Nepal, but the levels are higher in the current study [34]. *Shigella* MDR phenotypes are commonly associated with the carriage of plasmid-encoded drug resistance genes and horizontal plasmid transfer among clinical *Shigella* isolates ([35]. Additionally, the MDR *Shigella* emerge due to the AcrAB-TolC drug efflux pump, also involved in MDR among other Gram-negative bacteria such as *Escherichia coli* and *Enterobacter aerogenes* [36]. These suggest the mechanisms mediating drug resistance among the *Shigella* isolates recovered in children under five years in Banadir Hospital, Somalia.

### 4.1. Limitation

The findings may not represent the health situation for the entire Somali country because this was a single hospital-based study with a small sample size.

## 5. Conclusions

Our findings showed a high prevalence of shigellosis and *Shigella flexneri* as the predominant serogroup among children under five years of age in Banadir Hospital, Somalia. Ampicillin and trimethoprim-sulfamethoxazole are no longer appropriate treatments for shigellosis in children under five years in Banadir Hospital. MDR *Shigella* strains, including those resistant to ciprofloxacin and ceftriaxone, have emerged in Somalia, posing a public health challenge. Therefore, there is an urgent need for AMR surveillance and continuous monitoring to mitigate the further spread of the MDR *Shigella* strains in Banadir Hospital and beyond.

## Figures and Tables

**Table 1:** Sociodemographic characteristics of children and caregivers.

Category	Frequency	Percentage (%)
Gender		
Male	69	38.3
Female	111	61.7
Age group of children (months)		
0–12	67	37.2
>12–24	78	43.3
>24–36	19	10.6
>36–48	10	5.6
>48–60	6	3.3
Age group of caregivers		
<20	11	6.1
>20–25	28	15.6
>25–30	74	41.1
>30–35	43	23.9
>35–40	24	13.3
Number of children per household		
1	61	33.9
2	88	48.9
3	29	16.1
4	2	1.1
Marital status of the caregivers		
Single	2	1.1
Married	134	74.4
Divorced	26	14.4
Widowed/widower	18	10
Education level of the caregivers		
No formal education	132	73.3
Primary school dropout	37	20.6
Primary school	8	4.4
Secondary school and above	3	1.7
Number of people per household		
1-2	6	3.3
3-4	58	32.2
5-6	72	40
7-8	39	21.7
9-10	4	2.2
11-12	1	0.5

**Table 2:** Antibiotic susceptibility pattern of *Shigella* serogroups isolated.

Antibiotics	Susceptibility	*Shigella* serogroups		
		*Shigella dysenteriae* type 1, *n* (%)	*Shigella flexneri*, *n* (%)	*Shigella sonnei*, *n* (%)
AMP	S	0 (0)	0 (0)	0 (0)
	R	5 (100)	26 (100)	6 (100)
CIP	S	5 (100)	21 (80.8)	3 (50)
	R	0 (0)	5 (19.2)	3 (50)
SXT	S	0 (0)	0 (0)	0 (0)
	R	5 (100)	26 (100)	6 (100)
CRO	S	3 (60)	16 (61.5)	2 (33.3)
	R	2 (40)	10 (38.5)	4 (66.7)
TE	S	0 (0)	0 (0)	0 (0)
	R	5 (100)	26 (100)	6 (100)
AZM	S	5 (100)	26 (100)	6 (100)
	R	0 (0)	0 (0)	0 (0)

AMP: ampicillin (10 mg); TE: tetracycline (30 mg); CRO: ceftriaxone (30 mg); SXT: trimethoprim-sulfamethoxazole (co-trimoxazole) (25 mg); CIP: ciprofloxacin (5 mg); AZM: azithromycin (15 mg); S: susceptible; R: resistance.

**Table 3:** Risk factors for shigellosis among children under five years of age.

Category	Shigellosis		*χ*2 value	*p* value	COR (95%)	*p* value
	Present, *n* (%)	Absent, *n* (%)				
Gender						
Male	10 (5.6)	59 (41.3)	2.519	0.113	—	—
Female	27 (15)	84 (58.7)				
Age group of children (months)						
0–12	6 (16.2)	61 (42.7)	10.601	0.031^∗∗^	0.138	0.064
>12–24	21 (56.7)	57 (39.9)				
>24–36	7 (18.9)	12 (8.4)				
>36–48	2 (5.4)	8 (5.6)				
>48–60	1 (2.7)	5 (3.5)				
Age group of caregivers						
<20	1 (2.7)	10 (7.5)	4.477	0.345	—	—
>20–25	4 (10.8)	24 (18.0)				
>25–30	13 (35.1)	51 (38.3)				
>30–35	12 (32.4)	31 (23.3)				
>35–40	7 (18.9)	17 (12.8)				
Marital status of the caregivers						
Single	0 (0)	2 (1.4)	2.034	0.565	—	—
Married	25 (67.5)	109 (76.2)				
Divorced	7 (18.9)	19 (13.3)				
Widowed/widower	5 (13.5)	13 (9.1)				
Education level of the caregivers						
No formal education	29 (78.4)	103 (72.0)	13.515	0.004^∗∗^	0.058	0.439
Primary school dropout	2 (5.4)	35 (24.5)				
Primary school	4 (10.8)	4 (2.8)				
Secondary school and above	2 (5.4)	1 (0.7)				
Number of children per household						
1	11 (29.7)	50 (34.9)	1.010	0.908	—	—
2	20 (54.1)	68 (47.6)				
3	6 (16.2)	23 (16.1)				
4	0 (0)	2 (1.4)				
Number of people per household						
1-2	2 (5.4)	4 (2.8)	8.114	0.618	—	—
3-4	13 (35.1)	45 (31.5)				
5-6	15 (40.5)	57 (39.9)				
7-8	6 (16.2)	33 (23.1)				
9-10	1 (2.7)	3 (2.1)				
11-12	0 (0)	1 (0.7)				
Sources of family income						
Wages	9 (24.3)	32 (22.4)	1.158	0.282	—	—
Own business	12 (32.4)	65 (45.5)				
Agriculture	4 (10.8)	12 (8.4)				
No income	12 (32.4)	34 (23.8)				
Source of domestic water						
Public tap	12 (32.4)	35 (24.5)	1.857	0.395	—	—
Private tap	25 (67.6)	104 (72.7)				
Dam	0 (0)	1 (0.7)				
River	0 (0)	3 (2.1)				
Water storage						
Jerry cans	24 (64.9)	80 (55.9)	0.959	0.327	—	—
Tank	13 (35.1)	63 (44.1)				
Sanitary facility						
Communal pit latrine	15 (40.5)	42 (29.4)				
Private pit latrine	22 (59.5)	101 (70.6)				
Caregiver handwashing practice						
After visiting toilet	13 (35.1)	41 (28.7)	1.985	0.371	—	—
Before preparing/cooking food	2 (5.4)	19 (13.3)				
Before meals	22 (59.5)	83 (58.0)				
With soap and water	9 (24.3)	35 (24.5)	0.141	0.708	—	—
With water only	28 (75.7)	108 (75.5)				
Healthcare-seeking facility						
Private clinical/pharmacy	3 (8.1)	17 (11.9)	0.425	0.514	—	—
Public health facility	34 (91.9)	126 (88.1)				

^∗∗^shows significance.

## Data Availability

The primary data used to support the findings of this study are included within the article.
